# Perceptions and Experiences of Stakeholders on In‐Person and Virtual Visitation in Intensive Care Units: A Qualitative Study Protocol in China

**DOI:** 10.1002/hsr2.72361

**Published:** 2026-04-15

**Authors:** Li Liu, Shuqin Hong, Yan Gao, Wen Zhou, Xinyi Liu, Yulong Cao, Juan Yao, Xiaoling Tang, Xiuni Gan

**Affiliations:** ^1^ Department of Intensive Care Medicine The Second Affiliated Hospital of Chongqing Medical University Chongqing Chongqing China; ^2^ Department of Intensive Care Medicine Army Medical Center of PLA Chongqing Chongqing China; ^3^ Department of Intensive Care Medicine Chongqing General Hospital Chongqing Chongqing China; ^4^ Department of Nursing The Second Affiliated Hospital of Chongqing Medical University Chongqing Chongqing China

**Keywords:** family presence, intensive care unit, protocols & guidelines, qualitative research, virtual visiting

## Abstract

**Background and Aims:**

Existing research on in‐person and virtual visitation is predominantly quantitative, focusing on the effectiveness and safety of these practices in the intensive care unit (ICU). Significant differences may exist in the experiences of diverse stakeholder groups regarding ICU visitation across these modalities. Consequently, we aim to explore the lived experiences of patients, family members, and healthcare providers with in‐person or virtual visitation practices in the ICU.

**Methods:**

A descriptive phenomenological qualitative study will be conducted to gain an insight into the in‐person or virtual visitation practices of the patients, family members, and healthcare providers. Participants will be recruited through purposive sampling from ICUs of three large public hospitals in Southwest China, focusing on stakeholders (patients, family members, and healthcare providers) involved in ICU family visitation. Sociodemographic information of participants will be collected. In‐depth individual interviews will be conducted using a semi‐structured interview guide until data saturation is achieved. The study will adhere to the Consolidated Criteria for Reporting Qualitative Research (COREQ). Data analysis will follow Colaizzi's seven‐step phenomenological method. NVivo 15 will be employed for data management and analysis.

**Discussion:**

This study is expected to uncover variations in implementation, elucidate stakeholder perspectives, identify challenges inherent to each approach, and ultimately advance the development of family visitation practices in ICUs.

## 1 Introduction

The survival rate in the intensive care unit (ICU) has improved, but patients and their families remain at risk of experiencing significant psychological morbidity [1]. Research indicates that ICU survivors face a high risk of long‐term depressive symptoms [2], while family members frequently experience adverse psychological outcomes such as anxiety, depression, and post‐traumatic stress disorder [3], ultimately impairing their long‐term quality of life and health outcomes. Consequently, there is an urgent need for early interventions to mitigate the incidence of these conditions. Facilitating family presence in the ICU is a key strategy, as it promotes the communication between patients and their families, provides essential emotional support, helps to reduce the incidence of patient delirium and alleviate psychological distress among family members [4, 5].

In‐person visitation and virtual visitation are critical modalities for facilitating family presence in the ICU. Family presence is primarily facilitated through in‐person visitation, which is a crucial means of maintaining intimate connections and meeting emotional needs. However, such visitation is often restricted due to practical and safety concerns [6, 7, 8]. The COVID‐19 pandemic exacerbated these restrictions but also accelerated the widespread adoption of virtual visitation as an alternative [9, 10]. Nevertheless, there are significant differences in the standards for implementing virtual visitation across ICUs. Commonly targeted populations include alert and oriented patients or those at the end of life, while considerable debate persists regarding the appropriateness of virtual visitation for comatose or sedated patients [11]. Although virtual visitation has been shown to enhance the communication between patients and their families, alleviate psychological distress, and improve engagement in care or rehabilitation processes, it cannot fully substitute for in‐person visitation [8]. Challenges such as technological barriers, communication constraints, and staffing shortages further influence its effectiveness, contributing to current variability and uncertainty in practice [8].

Stakeholders—defined as individuals or groups responsible for or affected by family visitation decisions—primarily include patients, family members, and healthcare providers (clinicians, nurses, policymakers, and administrators), who play distinct yet critical roles in ICU family visitation practices [12]. Therefore, understanding stakeholders' experiences and needs is essential to address challenges across visitation modalities and improve family satisfaction [6, 13]. However, the existing quantitative studies on visitation have largely focused on describing the distribution and characteristics of ICU visiting policies [11, 14], identifying the factors influencing the implementation of visiting policy [10, 11], and evaluating the effects of visitation on patient physiological parameters and family psychological outcomes [4, 5, 15]. At the same time, existing qualitative inquiries have focused on clinical staff's perceptions of ICU visitation frameworks [16] and stakeholder perspectives (encompassing families and healthcare providers) regarding visitation paradigm shifts in the COVID‐19 context [17, 18, 19]. It is noteworthy that there is a lack of comparative analysis comparing the lived experiences of patients, family members, and healthcare providers across in‐person and virtual visitation modalities [20]. This important inquiry will facilitate the development of evidence‐based frameworks to guide the design and implementation of tailored family visitation programs.

## 2 Aims

We aim to systematically explore and characterize the lived experiences of ICU stakeholders in both in‐person and virtual visitation modalities. Specifically, this study seeks to:

Explore the lived experiences and perspectives of stakeholders across different visitation modalities.

Gain an in‐depth understanding of stakeholders' perceived challenges and expectations across different visitation modalities.

## 3 Methods

### 3.1 Study Design

We will conduct a descriptive phenomenological study [21, 22]. Descriptive phenomenology is well‐suited to exploring individuals' lived experiences in depth and revealing the essential nature of these experiences through their own perspectives. The Consolidated Criteria for Reporting Qualitative Research (COREQ) [23, 24] checklist will guide the methodological rigor and reporting of this study. A descriptive study design, guided by the stakeholder theory [12], will be employed to explore the knowledge, attitudes, and experiences of healthcare providers, patients, and family members in ICUs regarding in‐person and virtual visitation practices.

### 3.2 Setting and Sample

We will use maximum variation purposive sampling to recruit participants with different backgrounds, so that we can collect rich and detailed descriptions of both in‐person and virtual visitation experiences [25, 26]. Before recruitment, researchers will meet with ICU leadership to present the study and obtain informed consent. ICU managers will facilitate the recruitment of eligible healthcare providers, and eligible alert patients will be identified through collaboration with bedside nurses and in‐depth observation. Researchers will assist families with routine ICU visitation, including connecting virtual equipment and instructing on personal protective equipment (e.g., isolation gowns, masks). The researchers explicitly will not engage in any clinical communication, medical decision‐making, or emotional counseling. These interactions will facilitate a good relationship between researchers and patients' families, and help to recruit family members with substantive visitation experiences to obtain detailed insights into in‐person or virtual visitation practices. The estimated sample size will be determined by data saturation [25, 27].

Inclusion criteria for this study are as follows:

For patients: (1) Age ≥ 18 years; (2) ICU stay duration ≥ 48 h; (3) Alert and able to participate in the study during ICU admission or within 24 h post‐ICU discharge; (4) Ability to communicate effectively; (5) Experience of receiving in‐person or virtual visitation; (6) Provision of informed consent for voluntary participation.

For family members: (1) Age ≥ 18 years with the ability to communicate effectively; (2) Experience of participating in ICU visitation; (3) Provision of informed consent for voluntary participation.

For healthcare providers: (1) Currently employed in the ICU during the study period; (2) ≥ 1 year of work experience in the ICU; (3) Familiarity with ICU visitation policies; (4) Voluntary participation and willingness to engage in study procedures.

### 3.3 Data Collection

Two researchers of the research team, both experienced ICU nurses with expertise in qualitative research and extensive clinical experience, will conduct in‐depth interviews to explore the experiences of healthcare providers, patients, and families regarding in‐person or virtual visitation practices in ICUs. This approach aims to deeply understand the participants' internal feelings, cognitions, and meanings attributed to their visitation experiences. Prior to the interviews, researchers will explain the study, obtain informed consent, and conduct audio‐recorded interviews in a quiet, comfortable setting of the participant's choice. The study has begun in July 2025 and each interview will last approximately 15–40 min. Given that data collection have already begun, this article is presented as a post hoc protocol. Patient interviews may be conducted in one or two sessions to accommodate potential fatigue. Sociodemographic data of all participants will be collected during the interviews. Non‐verbal cues, such as tone, facial expressions, and gestures, will be documented in real time during and immediately following the interviews.

The interview guide has been developed based on existing literature, team members' clinical expertise, and methodological consultations. Pilot interviews were conducted with 2–3 participants from each stakeholder group (patients, family members, and healthcare providers). The interview guide was refined through pilot feedback and will be further adjusted as data collection proceeds. The interview guide is detailed in Table 1.

**Table 1:** Interview questions for patients, family members, and healthcare providers.

Patients	Family members	Healthcare providers
Could you elaborate on the most profound emotional experiences you have encountered during ICU hospitalization?	Could you describe your experience of involvement in family visitation? What will you do during these visits?	Could you delineate your specific clinical responsibilities within the current family visitation?
How do you evaluate the current visitation methods?	What difficulties or barriers have you encountered during recent family visitation experiences?	What systemic challenges or operational barriers have you encountered in implementing visitation procedures?
What advantages and limitations do you perceive between in‐person and virtual visitation?	What are your views on in‐person visitation? Please share your perspective on its strengths and limitations, along with the specific reasons behind your evaluation.	How would you assess the efficacy of the current visitation model?
How do you expect to carry out family visitation?	What kind of assistance do you hope to receive during the visitation? Why?	What is your analysis of the comparative clinical value between in‐person visitation and virtual visitation?
What would you like your family to do for you during the visitation?	What are your views on virtual visitation? Please share your perspective on its strengths and limitations, along with the specific reasons behind your evaluation.	What did the family do for the patient during the visitation? What is your opinions of the family's behavior?
	From your perspective, what constitutes an ideal visitation? Please consider the preferred time, frequency, length, number of visitors, and method.	In your view, what are the main risks involved in family visitation, and what are the underlying causes?
		Considering the existing visitation practices, what specific areas do you believe require improvement? Could you tell us your suggestions.

All qualitative interviews will be conducted using mobile device‐embedded audio recording systems to facilitate verbatim transcription and subsequent phenomenological analysis. To ensure ethical compliance, immediately after each interview, the audio file will be renamed with a unique, anonymous study ID (e.g., Case 1, Case 2, Case 3). The interview record will be prefixed with P, F, or H to distinguish patients, family members, and healthcare providers. The researchers will complete the transcription of the audio files within 48 h after the interview ends. The researchers will work under a confidentiality agreement. During the transcription process, the interviewer will conceal all potentially identifiable information of the research participants. Another researcher (not the interviewer) will further detect and remove any potential identifying information when conducting a second verification of the transcribed text. The anonymized transcript will serve as the sole version used for qualitative analysis. The original audio files will be stored separately on a password‐protected, encrypted server accessible only to the principal investigators and will be securely destroyed after a defined retention period.

### 3.4 Data Analysis

We will conduct a descriptive analysis of the data using the Colaizzi 7 analysis method [28]. The procedure will be as follows: two researchers who are trained in qualitative methods through formal coursework will independently perform coding using the Nvivo 15. This process will involve a close reading of the verbatim transcripts in conjunction with the on‐site notes to identify and extract significant statements. These statements will then be organized into meaning units, which will subsequently be categorized and clustered to form initial themes. Each theme will be carefully defined and described in detail. To ensure the trustworthiness of the analysis, the two researchers will compare their coding results and discuss any discrepancies to reach consensus. In cases where consensus cannot be achieved, a third researcher will be consulted. Finally, the derived thematic structure will be validated through member checking by returning it to a subset of participants for feedback.

### 3.5 Ethics and Dissemination

The Research Ethics Committee of the Second Affiliated Hospital of Chongqing Medical University approved this study (No.2024‐58‐1). This research plan will recruit research subjects face‐to‐face and collect data. Researchers will conduct interviews with family members either while they wait in the lounge or following the visit, ensuring that the visitation process is not disrupted. Given the critical condition of ICU patients, researchers will select those with relatively stable conditions and a strong willingness to express themselves as study participants. Researchers will inquire about willingness to be interviewed when patients feel fatigued, and interviews will be completed in one or two sessions based on their condition. The study will ensure voluntary participation and informed consent. Researchers will offer incentives in the form of small gifts to compensate for the participants' time.

Results will be published in peer‐reviewed journals and presented at scientific meetings.

### 3.6 Validity and Reliability/Rigour

To ensure trustworthiness, the researchers will establish prolonged engagement in the ICU to build rapport and elicit authentic accounts; employ reflexive journals, team discussions, and member checks to mitigate bias; and provide thick descriptions of the setting, participants, and visitation circumstances to support accurate interpretation. Finally, the results will be reported following the COREQ [23].

## 4 Discussion

Patients, their families and healthcare providers are important stakeholders in ICU family visitation. Currently, there are few studies exploring the viewpoints of patients, their families and healthcare providers on in‐person and virtual visitation. This study will explore and elucidate the lived experiences of patients, family members, and healthcare providers with in‐person and virtual visitation practices in the ICU, thereby providing evidence to improve family visitation implementation.

Building on these insights, the results of this study may (1) provide an empirical basis for developing or revising more flexible, inclusive, and actionable ICU visitation guidelines; (2) inform the design of targeted training materials for healthcare providers and support tools for families, based on the identified barriers and technological challenges; and (3) offer evidence for optimizing future technology platforms and clinical workflows, informed by a deeper understanding of the virtual visitation experience. This study is committed to contributing to the development of a more humane and sustainable model of ICU visitation.

The study will mainly select three large public hospitals in the southwest of China as sampling sites. The findings may not be representative of research subjects in other regions. However, the study will maximize sample diversity by including participants with different ages, genders, educational levels, etc., and will collect a wide range of experiences and views from stakeholders regarding in‐person and virtual visitation. Furthermore, researchers' involvement in ICU management may influence participants' perception of the visitation experience. However, during the assistance process, the researchers will follow the unit's unified and standardized protocols to minimize variation resulting from differing levels of researcher involvement.

In summary, by exploring the experiences of patients, family members, and healthcare providers in in‐person or virtual visitation practices, this study has the potential to provide an empirical basis for refining family visitation practices, improve the experience and health outcomes of patients and their families.

## Author Contributions

**Li Liu:** writing – review and editing, writing – original draft, methodology, investigation, formal analysis, data curation, conceptualization. **Shuqin Hong:** writing – review and editing, writing – original draft, methodology, investigation, formal analysis, conceptualization. **Yan Gao:** writing – review and editing, supervision, conceptualization, methodology, project administration. **Wen Zhou:** writing – review and editing, supervision, conceptualization, methodology, project administration. **Xinyi Liu:** investigation, methodology, formal analysis, supervision. **Yulong Cao:** investigation, methodology, formal analysis, supervision. **Juan Yao:** writing – review and editing, supervision, investigation, methodology, project administration. **Xiaoling Tang:** writing – review and editing, supervision, investigation, methodology, project administration. **Xiuni Gan:** writing – review and editing, supervision, methodology, project administration.

## Ethics Statement

This study was approved by the Research Ethics Committee of the Second Affiliated Hospital of Chongqing Medical University (No. 2024‐58‐1). The research subjects will voluntarily participate in the study and give informed consent to it. The data will remove all identifiable information of the research object to reduce the risk of its identification.

## Consent

The authors have nothing to report.

## Conflicts of Interest

The authors declare no conflicts of interest.

## Transparency Statement

The lead author Xiuni Gan affirms that this manuscript is an honest, accurate, and transparent account of the study being reported; that no important aspects of the study have been omitted; and that any discrepancies from the study as planned (and, if relevant, registered) have been explained.

## Data Availability

Data sharing not applicable to this article because no data sets are generated.

## References

[1] J. L. Tingey , N. A. Dasher , A. E. Bunnell , and A. J. Starosta , “Intensive Care‐Related Cognitive Impairment: A Biopsychosocial Overview,” PM&R 14, no. 2 (2022): 259–272. 35077003 10.1002/pmrj.12773

[2] Z. Du , X. Liu , Y. Li , et al., “Depressive Symptoms over Time Among Survivors After Critical Illness: A Systematic Review and Meta‐Analysis,” General Hospital Psychiatry 87 (2024): 41–47. 38306945 10.1016/j.genhosppsych.2023.12.008

[3] Z. Q. Cheng , B. Z. Zhang , J. Y. Xia , et al., “The Incidence and Risk Factors of Psychological Dysfunction in Patients’ Family Members After Intensive Care Unit: A Meta‐Analysis,” Military Nursing 40, no. 2 (2023): 102–106.

[4] R. Akbari , H. Karimi Moonaghi , S. R. Mazloum , and A. Bagheri Moghaddam , “Implementation of a Flexible Visiting Policy in Intensive Care Unit: A Randomized Clinical Trial,” Nursing in Critical Care 25, no. 4 (2020): 221–228. 31975479 10.1111/nicc.12499

[5] R. G. Rosa , J. A. S. Pellegrini , R. B. Moraes , et al., “Mechanism of a Flexible ICU Visiting Policy for Anxiety Symptoms Among Family Members in Brazil: A Path Mediation Analysis in a Cluster‐Randomized Clinical Trial,” Critical Care Medicine 49, no. 9 (2021): 1504–1512. 33870915 10.1097/CCM.0000000000005037

[6] J. Ning and V. Cope , “Open Visiting in Adult Intensive Care Units—A Structured Literature Review,” Intensive and Critical Care Nursing 56 (2020): 102763. 31668437 10.1016/j.iccn.2019.102763

[7] G. Frivold , A. S. Ågård , H. I. Jensen , et al., “Family Involvement in the Intensive Care Unit in Four Nordic Countries,” Nursing in Critical Care 27, no. 3 (2022): 450–459. 34405494 10.1111/nicc.12702

[8] K. A. Milner , “Evolution of Visiting the Intensive Care Unit,” Critical Care Clinics 39, no. 3 (2023): 541–558. 37230555 10.1016/j.ccc.2023.01.005

[9] S. Marmo and K. A. Milner , “From Open to Closed: COVID‐19 Restrictions on Previously Unrestricted Visitation Policies in Adult Intensive Care Units,” American Journal of Critical Care 32, no. 1 (2023): 31–41. 36175358 10.4037/ajcc2023365

[10] M. Brauchle , P. Nydahl , G. Pregartner , M. Hoffmann , and M. M. Jeitziner , “Practice of Family‐Centred Care in Intensive Care Units Before the COVID‐19‐pandemic: A Cross‐Sectional Analysis in German‐Speaking Countries,” Intensive and Critical Care Nursing 68 (2022): 103139. 34750041 10.1016/j.iccn.2021.103139 PMC8421104

[11] L. Rose , L. Yu , J. Casey , et al., “Communication and Virtual Visiting for Families of Patients in Intensive Care During the COVID‐19 Pandemic: A UK National Survey,” Annals of the American Thoracic Society 18, no. 10 (2021): 1685–1692. 33617747 10.1513/AnnalsATS.202012-1500OC PMC8522289

[12] T. W. Concannon , S. Grant , V. Welch , et al., “Practical Guidance for Involving Stakeholders in Health Research,” Journal of General Internal Medicine 34, no. 3 (2019): 458–463. 30565151 10.1007/s11606-018-4738-6 PMC6420667

[13] X. P. Jiao and R. Y. Liu , “Research Progress on Visiting Modes in Intensive Care Unit,” Chinese Nursing Research 35, no. 5 (2021): 851–855.

[14] T. Langer , F. C. Depalo , C. Forlini , et al., “Communication and Visiting Policies in Italian Intensive Care Units During the First COVID‐19 Pandemic Wave and Lockdown: A Nationwide Survey,” BMC Anesthesiology 22, no. 1 (2022): 187. 35710331 10.1186/s12871-022-01726-1 PMC9203262

[15] J. M. B. de Souza , A. P. Miozzo , R. da Rosa Minho Dos Santos , et al., “Long‐Term Effects of Flexible Visitation in the Intensive Care Unit on Family Members' Mental Health: 12‐Month Results From a Randomized Clinical Trial,” Intensive Care Medicine 50, no. 10 (2024): 1614–1621. 39172240 10.1007/s00134-024-07577-3

[16] K. A. Milner , S. Marmo , and S. Goncalves , “Implementation and Sustainment Strategies for Open Visitation in the Intensive Care Unit: A Multicentre Qualitative Study,” Intensive and Critical Care Nursing 62 (2021): 102927. 32855008 10.1016/j.iccn.2020.102927 PMC7444949

[17] L. Rose , T. Graham , A. Xyrichis , et al., “Family Perspectives on Facilitators and Barriers to the Set Up and Conduct of Virtual Visiting in Intensive Care During the COVID‐19 Pandemic: A Qualitative Interview Study,” Intensive and Critical Care Nursing 72 (2022): 103264. 35672211 10.1016/j.iccn.2022.103264 PMC9114263

[18] N. R. Kennedy , A. Steinberg , R. M. Arnold , et al., “Perspectives on Telephone and Video Communication in the Intensive Care Unit During Covid‐19,” Annals of the American Thoracic Society 18, no. 5 (2021): 838–847. 33181033 10.1513/AnnalsATS.202006-729OC PMC8086546

[19] J. A. Greenberg , S. Basapur , T. V. Quinn , et al., “Challenges Faced by Families of Critically Ill Patients During the First Wave of the COVID‐19 Pandemic,” Patient Education and Counseling 105, no. 2 (2022): 297–303. 34507866 10.1016/j.pec.2021.08.029 PMC8393512

[20] K. M. Fiest , K. D. Krewulak , L. C. Hernández , et al., “Evidence‐Informed Consensus Statements to Guide COVID‐19 Patient Visitation Policies: Results From a National Stakeholder Meeting,” Canadian Journal of Anesthesia/Journal canadien d'anesthésie 69, no. 7 (2022): 868–879. 10.1007/s12630-022-02235-y PMC8970637 35359262

[21] S. Shorey and E. D. Ng , “Examining Characteristics of Descriptive Phenomenological Nursing Studies: A Scoping Review,” Journal of Advanced Nursing 78, no. 7 (2022): 1968–1979. 35405036 10.1111/jan.15244 PMC9320962

[22] I. Korstjens and A. Moser , “Series: Practical Guidance to Qualitative Research. Part 2: Context, Research Questions and Designs,” European Journal of General Practice 23, no. 1 (2017): 274–279. 29185826 10.1080/13814788.2017.1375090 PMC8816399

[23] A. Tong , P. Sainsbury , and J. Craig , “Consolidated Criteria for Reporting Qualitative Research (COREQ): a 32‐item Checklist for Interviews and Focus Groups,” International Journal for Quality in Health Care 19, no. 6 (2007): 349–357. 17872937 10.1093/intqhc/mzm042

[24] N. Buus and A. Perron , “The Quality of Quality Criteria: Replicating the Development of the Consolidated Criteria for Reporting Qualitative Research (COREQ),” International Journal of Nursing Studies 102 (2020): 103452. 31726311 10.1016/j.ijnurstu.2019.103452

[25] A. Moser and I. Korstjens , “Series: Practical Guidance to Qualitative Research. Part 3: Sampling, Data Collection and Analysis,” European Journal of General Practice 24, no. 1 (2018): 9–18. 29199486 10.1080/13814788.2017.1375091 PMC5774281

[26] J. Shi , C. Zhang , Q. Zhao , X. Zhang , L. Guo , and T. Jia , “Experience of Patients With Diabetic Retinopathy: A Qualitative Study,” Journal of Advanced Nursing 79, no. 5 (2023): 1789–1798. 36218198 10.1111/jan.15457

[27] M. Hennink and B. N. Kaiser , “Sample Sizes for Saturation in Qualitative Research: A Systematic Review of Empirical Tests,” Social Science & Medicine 292 (2022): 114523. 34785096 10.1016/j.socscimed.2021.114523

[28] M. N. Alhalabi , I. A. Khalaf , R. S. Zeilani , H. A. Bawadi , A. S. Musa , and A. J. Nashwan , “Palliative Care Needs of Jordanian Women's Experience of Living With Stroke: A Descriptive Phenomenological Study,” BMC Palliative Care 22, no. 1 (2023): 106. 37507696 10.1186/s12904-023-01216-2 PMC10375733

